# Optimization of handheld spectrally encoded coherence tomography and reflectometry for point-of-care ophthalmic diagnostic imaging

**DOI:** 10.1117/1.JBO.29.7.076006

**Published:** 2024-07-24

**Authors:** Jacob J. Watson, Rachel Hecht, Yuankai K. Tao

**Affiliations:** Vanderbilt University, Department of Biomedical Engineering, Nashville, Tennessee, United States

**Keywords:** optical coherence tomography, multimodal imaging, ophthalmology, retinal imaging, diagnostics, point-of-care

## Abstract

**Significance:**

Handheld optical coherence tomography (HH-OCT) systems enable point-of-care ophthalmic imaging in bedridden, uncooperative, and pediatric patients. Handheld spectrally encoded coherence tomography and reflectometry (HH-SECTR) combines OCT and spectrally encoded reflectometry (SER) to address critical clinical challenges in HH-OCT imaging with real-time *en face* retinal aiming for OCT volume alignment and volumetric correction of motion artifacts that occur during HH-OCT imaging.

**Aim:**

We aim to enable robust clinical translation of HH-SECTR and improve clinical ergonomics during point-of-care OCT imaging for ophthalmic diagnostics.

**Approach:**

HH-SECTR is redesigned with (1) optimized SER optical imaging for *en face* retinal aiming and retinal tracking for motion correction, (2) a modular aluminum form factor for sustained alignment and probe stability for longitudinal clinical studies, and (3) one-handed photographer-ergonomic motorized focus adjustment.

**Results:**

We demonstrate an HH-SECTR imaging probe with micron-scale optical-optomechanical stability and use it for *in vivo* human retinal imaging and volumetric motion correction.

**Conclusions:**

This research will benefit the clinical translation of HH-SECTR for point-of-care ophthalmic diagnostics.

## 1 Introduction

Optical coherence tomography (OCT) is the gold standard for non-invasive ophthalmic imaging and enables depth-resolved visualization of retinal microstructures.^1^ Most commercial OCT systems utilize a slit-lamp design that requires patients to fixate and be imaged upright, which precludes imaging of bedridden, uncooperative, and pediatric patients. The iVue (Optovue Inc., Fremont, California, United States) and the ENVISU R-Class (Leica Microsystems, GmbH, Wetzlar, Germany) are the only commercially available systems with a handheld OCT (HH-OCT) probe for point-of-care imaging in these patient populations. However, slow imaging speeds (<100  kHz) leave these systems susceptible to artifacts from involuntary physiological motion, including patient eye movements and photographer hand tremors, during data acquisition.^2^^–^^5^ HH-OCT research prototypes have moved to higher imaging speeds to minimize these artifacts,^6^^–^^10^ but higher speeds result in inherent performance trade-offs by reducing signal-to-noise ratio.^11^^,^^12^ Higher imaging speeds additionally reduce HH-OCT angiography sensitivity (OCTA),^13^ which requires repeated frames to be imaged with enough time delay that scatterers are sufficiently decorrelated to resolve blood flow dynamics.^14^^–^^16^

High-speed *en face* imaging can address these trade-offs using fundus feature registration for retinal tracking and OCT motion correction. Real-time *en face* retinal previews also benefit the alignment of OCT volumes to retinal regions of interest (ROIs) as compared to OCT B-scans or pupil/iris tracking.^5^^,^^8^^,^^10^ Retinal tracking and aiming have been demonstrated as part of multimodal systems that combine OCT with high-speed *en face* imaging technologies, such as scanning laser ophthalmoscopy (SLO). However, these systems (1) are unable to perform simultaneous multimodal image acquisition, (2) lack multimodal spatial co-registration, and/or (3) have bulky and cumbersome form factors.^17^^–^^22^

An additional requirement for high-quality point-of-care retinal OCT imaging is ocular focus adjustment. Defocus occurs from changes in corneal curvature and abnormal eye length, which causes light to focus imperfectly on the retina. Consequently, defocus impacts retinal image quality in all ametropic patients. Focus adjustments are essential for imaging severe myopes, who are at higher risk of irreversible vision loss from cataracts, glaucoma, retinal detachment, and macular degeneration.^23^ Premature infants also require focus adjustments for retinopathy of prematurity screening due to changes in ocular power and axial eye length during the neonatal period.^24^^,^^25^ Additionally, focus adjustments are critical for vascular contrast in OCTA, which degrades significantly with defocus.^26^^,^^27^

Our research group has introduced spectrally encoded coherence tomography and reflectometry (SECTR) to concurrently overcome multimodal imaging limitations for point-of-care ophthalmic imaging.^5^^,^^28^^,^^29^ SECTR integrates spectrally encoded reflectometry (SER) with swept-source OCT for spatiotemporally co-registered *en face* and cross-sectional imaging with reduced system complexity. We have previously demonstrated a handheld SECTR (HH-SECTR) imaging probe and synergistic algorithms that enabled (1) OCT/OCTA motion correction with complementary SER retinal tracking and (2) real-time *en face* SER retinal aiming for pupil alignment and OCT volume alignment to retinal ROIs.^5^ However, this HH-SECTR probe could not be clinically translated due to (1) low SER optical power throughput and alignment reproducibility; (2) weak mechanical stiffness of its rapid-prototyped resin body, which resulted in poor sustained alignment and significant field of view (FOV) drift during operation; and (3) a manual ophthalmic lens focus adjustment mechanism that required cumbersome two-handed photographer operation.

In this work, we present a clinically robust HH-SECTR system with (1) a high-power SECTR optical engine and throughput-optimized SER imaging optics for improved *en face* imaging, (2) an aluminum probe chassis with micron-scale stability and sustained alignment, and (3) ergonomic motorized focusing for one-handed photographer imaging and focus adjustment. We demonstrate theoretical and experimental validation of the micron-scale mechanical stability of the presented HH-SECTR probe. We also demonstrate *in vivo* human retinal imaging with volumetric motion correction and one-handed focus adjustment with our new HH-SECTR imaging system.

## 2 Materials and Methods

### 2.1 SECTR Engine and Data Acquisition

**Fig. 1:**
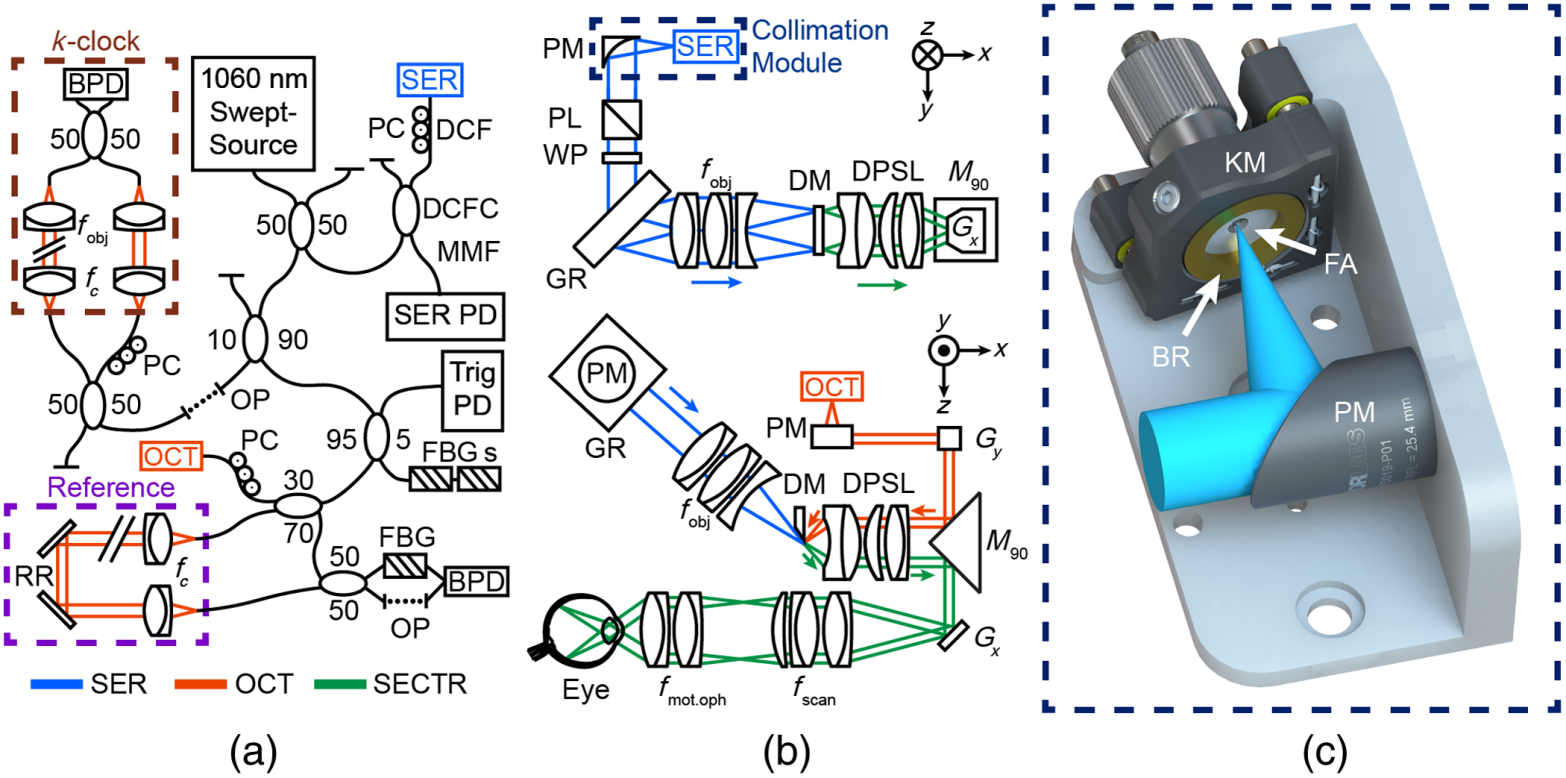
HH-SECTR system schematic. (a) SECTR optical engine. BPD, balanced photodetector; DCF, double-clad fiber; DCFC, double-clad fiber coupler; FBG, fiber Bragg grating; MMF, multimode fiber; OP, optical fiber patch cord; PC, polarization controller; PD, photodiode; RR, retroreflector. (b) HH-SECTR imaging optics. DM, D-shaped mirror; DPSL, double-pass scan lens; $f_{\mathrm{obj}}$, SER objective lens; $f_{\mathrm{mot}.\mathrm{oph}}$, motorized ophthalmic lens; $f_{\text{scan}}$, scanning lens; GR, diffraction grating; $G_{x,y}$, galvanometer; $M_{90}$, 90-deg knife-edge prism mirror; PL, polarizer; PM, parabolic mirror collimator; WP, quarter wave-plate. The dashed navy box indicates the SER collimation module. (c) SER collimation bench module. KM, kinematic mount; BR, brass mounting ring; FA, fiber adaptor; PM, parabolic mirror collimator.

A new SECTR engine was developed to accommodate a 400 kHz, 1051±46  nm, 68% duty cycle, bidirectional swept-source laser (Axsun, Saint-Hubert, Canada) with 52 mW output power [Fig. 1(a)]. This provided more optical power than our previous engine, and different coupling ratios were implemented to route more optical power to the SER channel for improved retinal contrast.^5^^,^^28^ A 1051.654±0.01  nm fiber Bragg grating (FBG) with 99.97% reflectivity (O-E Land Inc., Montreal, Canada) and path length matched single-mode fiber were added to OCT balanced photodiode inputs for bidirectional sweep alignment and sweep-to-sweep drift compensation to account for inherent trigger fluctuations in post-processing.^28^^,^^30^ SER was detected using a 240-MHz avalanche photodiode (A-CUBE-I200-240, Laser Components, Bedford, New Hampshire, United States) with reduced noise and improved dynamic range compared to our previous detector.^5^^,^^28^

Our previously described engine electronics and optical components for k-clocking and triggering were optimized for a unidirectional source and were redesigned to accommodate the new bidirectional source.^5^^,^^28^ 2.25% of the engine power was coupled to two FBGs (O/E Land Inc.) with corresponding wavelengths at the beginning (1007.934±0.01  nm) and end (1095.132±0.01  nm) of the laser sweep bandwidth. FBG reflections were detected with the same avalanche photodiode as the previous system^5^^,^^28^ and conditioned with a flip flop (CD74ACT109E, Texas Instruments, Dallas, Texas, United States) and operational amplifier (LMH6629, Texas Instruments) to create a 400 kHz transistor-to-transistor logic trigger signal with the rising edge synchronized to the beginning of both the forward and backward sweeps for bidirectional triggering. Inherent inter-sweep noise from the bidirectional laser source required modification of previous k-clocking electronics to minimize data acquisition artifacts.^5^^,^^28^ The Mach Zehnder interferometer was set to a 1.5 GHz fringe frequency, detected (APD481-AC, ThorLabs, Newton, New Jersey, United States), high-pass filtered (SHP-400 +, Mini-Circuits, Brooklyn, New York, United States), conditioned with a comparator (ADCMP565, Analog Devices, Wilmington, Massachusetts, United States), and amplified (ZKL-2+, ZX60-H242+, Mini-Circuits) to allow each 400 kHz laser sweep to be sampled with up to 1792 samples. C++ software was previously developed for data acquisition, real-time display, and archiving.^28^ Optical power differences between forward and backward sweeps were compensated using histogram-based intensity normalization in post-processing.

### 2.2 SER Imaging

SER achieves high *en face* frame rates (>200  Hz) using spectrally encoded line illumination in one dimension and scanning in the orthogonal (fast-axis) dimension. However, direct detection of weakly backscattering retinal tissue with our 400 kHz swept-source system makes SER retinal image quality and contrast highly sensitive to optical losses—much more so than OCT. To increase retinal contrast in this redesign, SER illumination power was increased by 136% at the pupil plane compared to our previous HH-SECTR probe by using the higher-power source optical engine and optimizing the alignment and optical power throughput of SER relay optics.^5^ SER illumination was collimated to a 9.65-mm beam diameter using an off-axis parabolic mirror collimator (MPD019-P01, ThorLabs) to reduce power loss as compared to the previous multi-element refractive collimation optics (10-mm beam diameter).^5^ SER imaging optics were benchtop-aligned to the first diffraction order of the grating to ensure maximum optical power throughput [Fig. 1(b), GR; PING-1379-422, Ibsen Photonics, Farum, Denmark].

SER detection with an avalanche photodiode is easily saturated by reflections in the optical path and sample, limiting the usable detector gain and digitizer dynamic range. To reduce back-coupled specular reflections, circularly polarized SER illumination and cross-polarization detection were implemented by adding a linear polarizer (GL15-C26, ThorLabs) and quarter waveplate (AQWP10M, ThorLabs) between the SER collimator and diffraction grating. Polarization controllers on the double-clad fiber were used to align the SER illumination polarization state to the linear polarizer orientation to maximize optical power throughput [Fig. 1(a)].^31^

### 2.3 SER Alignment

SER fiber alignment reproducibility was a critical limitation of our previous HH-SECTR probe because the double-clad fiber is terminated with a length of custom angle-polished no-core fiber used to minimize end-face reflection coupling into the multimode inner cladding.^5^ Tolerances in the polish angle during fabrication make SER fiber alignment using standard fixed fiber-to-free-space adapters impossible and require axial, tip, tilt, and rotational degrees of freedom (DOF). This difficulty is compounded by the lightweight, compact, and alignment-robust form-factor requirements for handheld systems, which are critical for clinical ease of use and sustained alignment for imaging.

A custom collimation fiber-bench module was designed for maximum fiber-coupling to the parabolic mirror collimator to ensure high SER alignment reproducibility in a compact form factor [Fig. 1(c)]. A narrow-key bulkhead fiber adaptor (HAFC2, ThorLabs) was fitted to a custom brass mounting ring and housed in a 0.5-in. kinematic mount (MK05, ThorLabs). The mounting ring facilitates axial and rotation DOFs, and the kinematic provides tip and tilt DOFs. A custom aluminum bench houses the parabolic mirror collimator and positions the kinematic angularly to the optical axis to compensate for the custom polish angle of the SER fiber. The parabolic mirror is centered between two through-holes spaced 1 in. apart on the bench, which facilitates easy external alignment and collimation assessment on a standard 1-in. breadboard. The bench has a rotational DOF centered under the parabolic mirror to ensure collimated beam alignment to downstream optics when inserted into the full optical system.

### 2.4 Limitations of Resin-Based Rapid-Prototyping

Many research groups, including our own, have utilized rapid-prototyped bodies for lightweight handheld OCT probes.^7^^–^^10^^,^^32^ While rapid prototyping allows compact hardware design with complex geometries, our previous HH-SECTR probe suffered from weak mechanical stiffness from its resin body (VeroBlue resin, Stratasys, Ltd., Eden Prairie, Minnesota, United States), which resulted in poor sustained alignment and significant FOV drift during operation.^5^ We investigated the stability of our resin-based probe with finite element analysis (FEA) on the mechanical assembly (SolidWorks Simulation, Dassault Systemès, Vélizy-Villacoublay, France). The FEA was set up with the lens tube in the probe handgrip as the fixation point to mimic use for supine imaging (Fig. 3, green arrows). Some off-the-shelf component models were FEA incompatible, so custom models were replicated as close to manufacturer specification as possible for compatibility.

This setup was simulated under the force of gravity to analyze overall scan head displacement. FEAs were performed with different orientations of gravity to investigate volumetric mechanical displacement (X, Y, and Z). FEA results indicate up to 41  μm of displacement across the body of the resin-based probe [Figs. 3(a), 3(f), and 3(k)], which is a four-fold displacement above positional tolerances for high-precision optics (<10  μm).^33^ We attribute the challenges in sustained probe alignment to mechanical displacement caused by the low modulus of elasticity of the resin-based probe body (2.2 GPa; VeroBlue, Stratasys). While seemingly small, this amount of mechanical displacement could result in degraded imaging performance from (1) reduced power coupling into the first diffraction order of the SER grating, (2) OCT and SER foci clipping at the double-pass scan lens (DPSL) intermediate image plane (IIP), and (3) compounding displacements across the body resulting in ophthalmic scan and relay lens clipping while scanning. Based on FEA comparisons with experimental stability measurements (Sec. 3.3), we hypothesize that the actual beam displacements would have been significantly higher.

### 2.5 Optomechanical Design and Prototyping

**Fig. 2:**
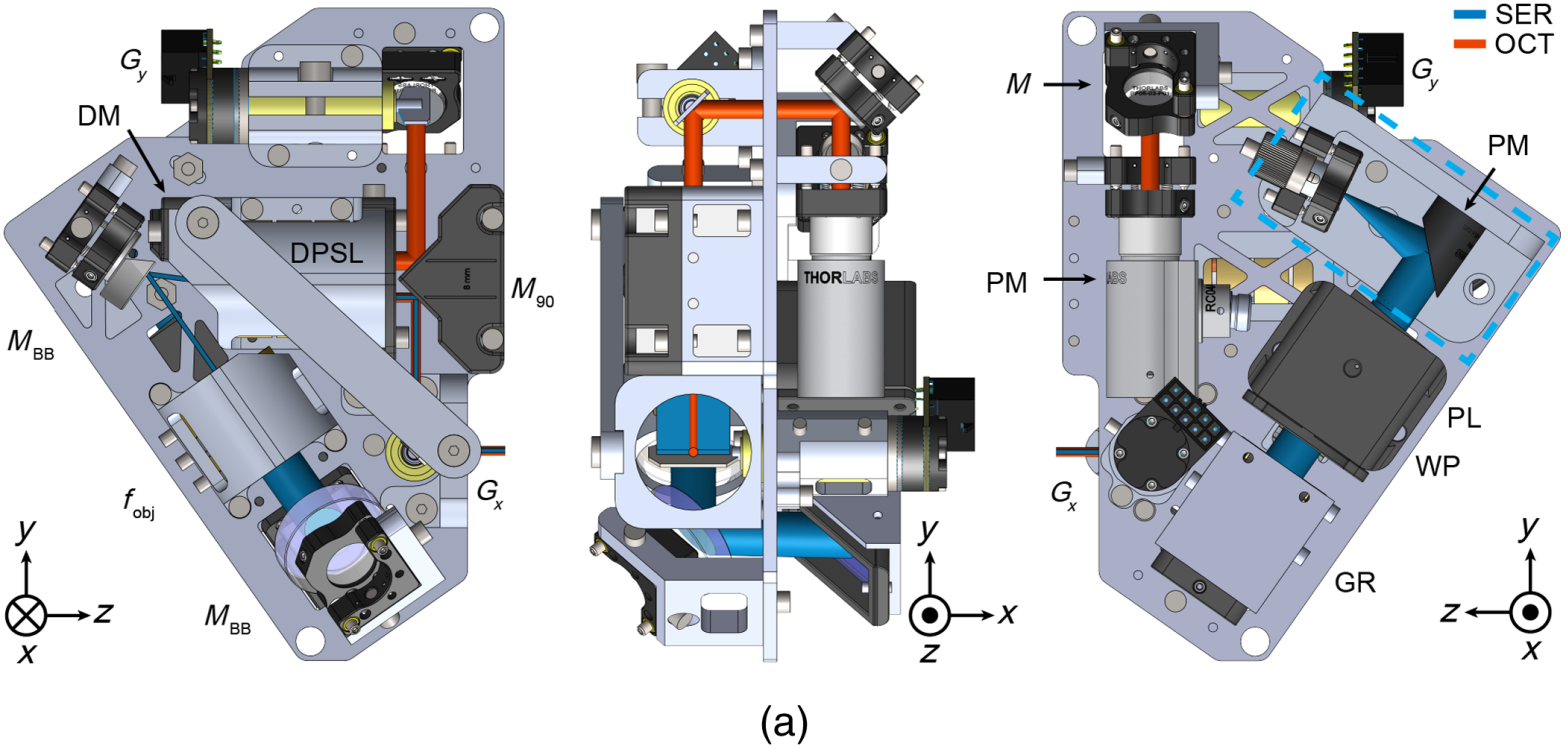
(a) HH-SECTR modular aluminum scan head with imaging optics. DM, D-shaped mirror; DPSL, double-pass scan lens; $f_{\mathrm{obj}}$, SER objective lens; GR, diffraction grating; $G_{x,y}$, galvanometer; M, silver turn mirror; $M_{BB}$, broadband dielectric mirror; $M_{90}$, 90-deg knife-edge prism mirror; PL, polarizer; PM, parabolic mirror collimator; WP, quarter wave-plate. The dashed box indicates the SER collimation fiber-bench module (Video 2, MP4, 6.18 MB [URL: https://doi.org/10.1117/1.JBO.29.7.076006.s2]).

An aluminum scan head chassis was designed to replace the rapid-prototyped housing to ensure HH-SECTR probe stability for robust point-of-care imaging (Fig. 2). 6061-T6 aluminum offers good machinability, a high strength-to-weight ratio, and relatively low density compared to other metals and provides more than a 30-fold increase in modulus of elasticity over the previously used rapid-prototyped resin body material (68.9 GPa versus 2.2 GPa). While utilizing aluminum avoids resin-prototyping limitations, it requires considerations for machinability and precision alignment. A modular design with a dual-sided baseplate and drop-in optical mounts and optomechanics enables individual component machining and precision alignment with dowel pins. This proves ideal for prototyping reproducibility and reduces geometric complexity for machining.

Scan head mechanical design was performed in SolidWorks (Dassault Systemès). The ophthalmic scanning and relay lenses were positioned to fit vertically in the photographer’s hand, with the scan head’s center of gravity positioned over the hand and wrist for ergonomic supine imaging. A knife-edge prism mirror (MRAK25-P01, ThorLabs) was added to orient the galvanometer scanning paths for overall scan head compactness. The dielectric D-shaped pickoff mirror used to co-align SER and OCT in the previous probe design was replaced with a silver knife-edge pickoff mirror [Fig. 1(a), DM; 36-137, Edmund Optics, Barrington, New Jersey, United States] to avoid introducing additional chromatic dispersion to OCT.^5^ Since the alignment of the SER polarizer and quarter wave plate was not critical for imaging performance, they were housed in a rapid-prototyped mount to overcome additional geometrical machining challenges. To facilitate onboard differential alignment capabilities, the reflective collimator (RC04APC-P01, ThorLabs) and silver fold mirror were kinematic-mounted for OCT, and the two dielectric fold mirrors were kinematic-mounted for SER. The SER objective lens mount (Fig. 2, $f_{\mathrm{obj}}$) provided an axial DOF to ensure SER and OCT foci alignment. Relief cuts on select mounts and the baseplate reduced weight without compromising mechanical stability. Custom brass spacers maintained precise alignment of the DPSL and SER objective lens groups. Set-screws and rapid-prototyped caps pressure-retained select components in their respective mounts. A rapid-prototyped enclosure protects the scan head from dust and ambient light and can be replaced as necessary for clinical sterility. The assembled HH-SECTR scan head measured 8.2  cm×14.2  cm×10.6  cm (width (X) × height (Y) × length (Z)) and weighed 748 g. The machined mounts and baseplate comprised 60.2% (450 g) of the scan head’s weight, and weight-relieving reduced overall weight by 12.1% (61.7 g).

### 2.6 Assessment of Aluminum-Based Hardware

**Fig. 3:**
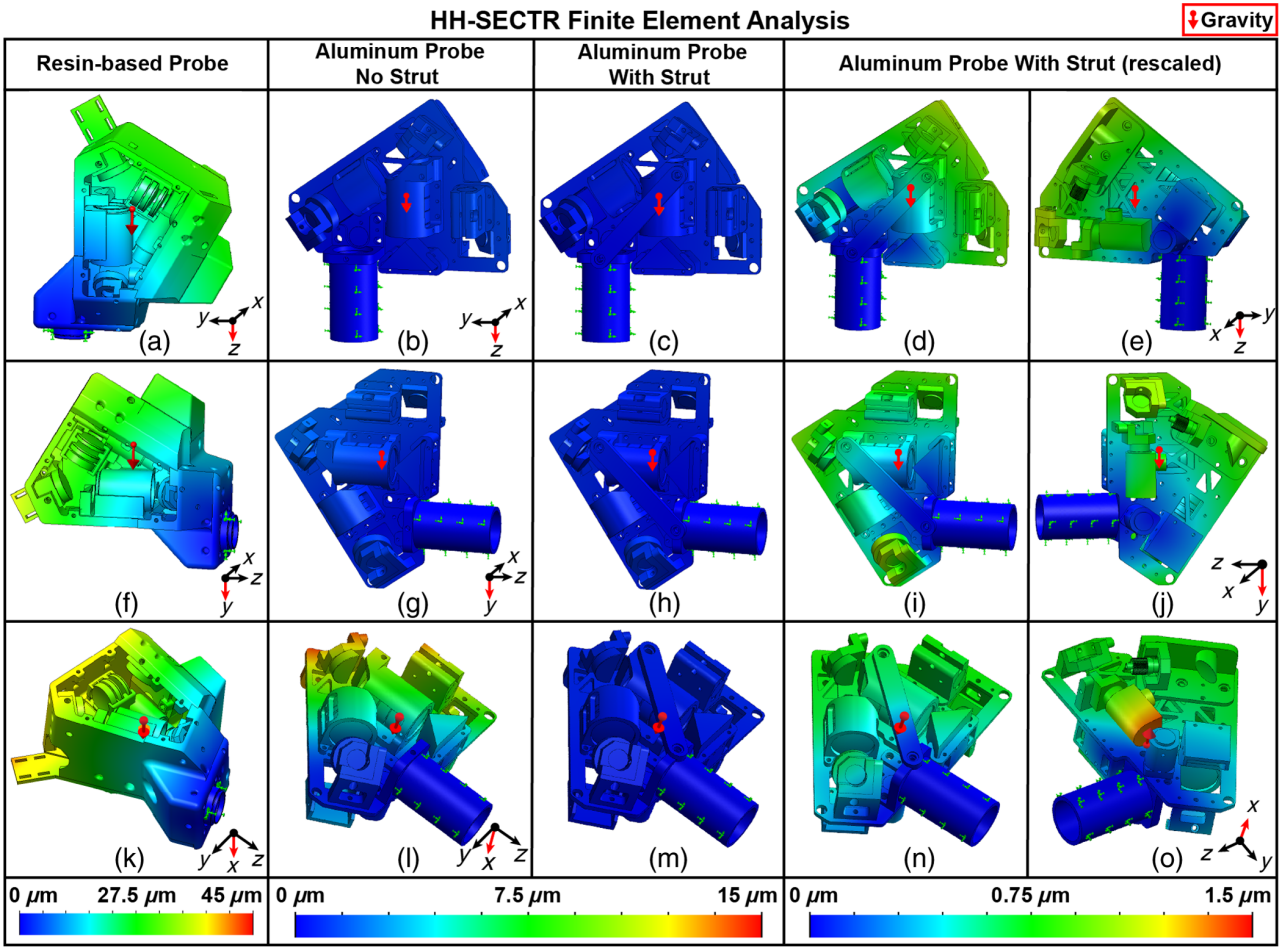
HH-SECTR finite element analysis. Gravity (red arrow) is oriented in the Z-axis for (a)–(e), Y-axis for (f)–(j), and X-axis for (k)–(o). The first column shows resin-based probe displacement where displacement is mapped up to 45  μm. The second (b), (g), (l) and third (c), (h), (m) columns show aluminum probe displacement without and with the strut, respectively, where displacement is mapped up to 15  μm. The fourth (d), (i), and (m) and fifth (e), (j), (o) columns display opposing sides of the optimized aluminum probe with the strut, where displacement is mapped up to 1.5  μm.

FEAs were performed on the aluminum scan head assembly to evaluate and compare mechanical stability to the resin-based probe (Fig. 3). The FEAs on the aluminum scan head assembly were set up similarly to the resin-based FEA setups, with the scanning lens tube as the point of fixation and identical orientations of gravity. The X-axis analysis indicated 14.1  μm of displacement across the scan head due to baseplate flexing [Fig. 3(l)], which is still greater than acceptable precision tolerances.^33^ Weight relief cuts had a negligible impact on overall displacement. Increasing baseplate thickness was an undesirable solution because of the additional probe size and weight. Machining the baseplate from a more rigid material was undesirable because of added weight, increased machining complexity, and little reduction in flexing compared to increasing baseplate thickness. An optimized strut spanning most of the scan head was added to reduce baseplate flexing with minimal complexity and additional weight [Figs. 3(c)–3(e), 3(h)–3(j), and 3(m)–3(o)]. The lens tube and DPSL mounts spanned most of the scan head body and provided convenient attachment points for the strut with minimal added design complexity. Strut angle and thickness were manually adjusted to balance beam displacement reduction and additional scan head weight. The addition of the strut reduced overall baseplate flexing to approximately 1.0  μm. With the strut, the most displacement is observed across the stainless-steel OCT reflective collimator (1.4  μm) in the X-axis FEA [Fig. 3(o)], which is the heaviest individual component (65 g) on the scan head and is suspended from a kinematic mount (MK05, ThorLabs). FEA results of the optimized aluminum scan head with the strut show single-micrometer mechanical stability, which is a 40-fold decrease in mechanical displacement compared to the resin-based probe and within positional tolerance metrics.

### 2.7 Experimental Validation

**Fig. 4:**
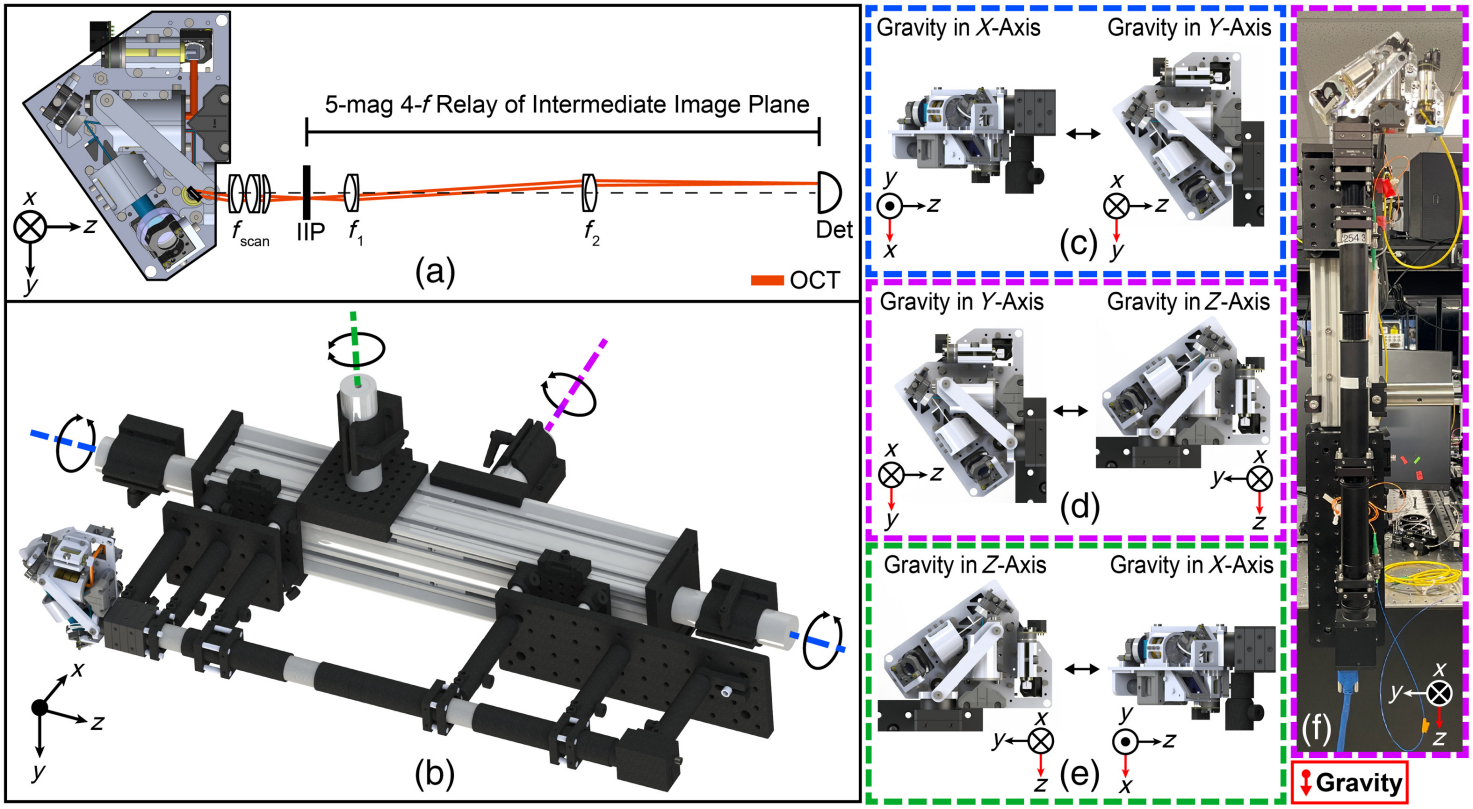
Experimental setup to quantify HH-SECTR scan head stability. (a) Optical 4-f relay to magnify HH-SECTR IIP for quantification of OCT beam displacement. (b) CAD of the experimental setup. The blue, purple, and green dotted lines indicate the corresponding axes for pivoting the setup to reorient gravity among the (c) X- and Y-axes, (d) Y- and Z-axes, and (e) Z- and X-axes, respectively. (f) Image of the experimental setup for the Y- and Z-axes. The direction of gravity is indicated by the red arrow.

A 5×-magnification 4-f relay was constructed to validate the aluminum scan head’s theoretical stability by magnifying OCT beam displacement at the IIP. Beam displacements were quantified with 1.1  μm accuracy using a NIR CMOS detector (5.5  μm pixel size, Grasshopper3, FLIR, Wilsonville, Oregon, United States) (Fig. 4) at the image plane of the relay. Since the OCT path spans the majority of the scan head, the compounding effects of baseplate flexing or other mechanical instabilities across the scan head are well represented by OCT IIP beam displacement. Several locked cage plates secured the scanning lens tube of the aluminum scan head to the relay to replicate FEA fixation conditions. The setup was placed on 1.5-in. steel pivots and rotated between the FEA orientations [Fig. 4(b)]. The beam position was recorded after each rotation. This procedure was repeated four times with and without the strut on the scan head, and with a single-mode optical fiber in a fixed free-space adaptor in the relay’s object plane to isolate displacements inherent to the setup and provide a baseline for experimental error.

An additional freehand stability experiment was performed to simulate and assess stability during point-of-care clinical use for supine imaging of the retinal periphery. The detector was mounted to the ophthalmic scan lens tube at the IIP, and the scan head was pivoted by hand by a volunteer photographer through 40 deg off-axis tilts. This freehand experiment was repeated with and without the strut on the scan head. Feedback to the volunteer photographer using real-time gyroscopic data from an inertial measurement unit (Bosch, BNO055, Gerlingen, Germany) ensured experimental consistency in pivoting the scan head by hand. OCT beam displacement was quantified for both stability experiments with rigid translation registration by computing the cross-correlation in the spatial frequency domain via discrete Fourier transform.^34^

### 2.8 Motorized Focusing

**Fig. 5:**
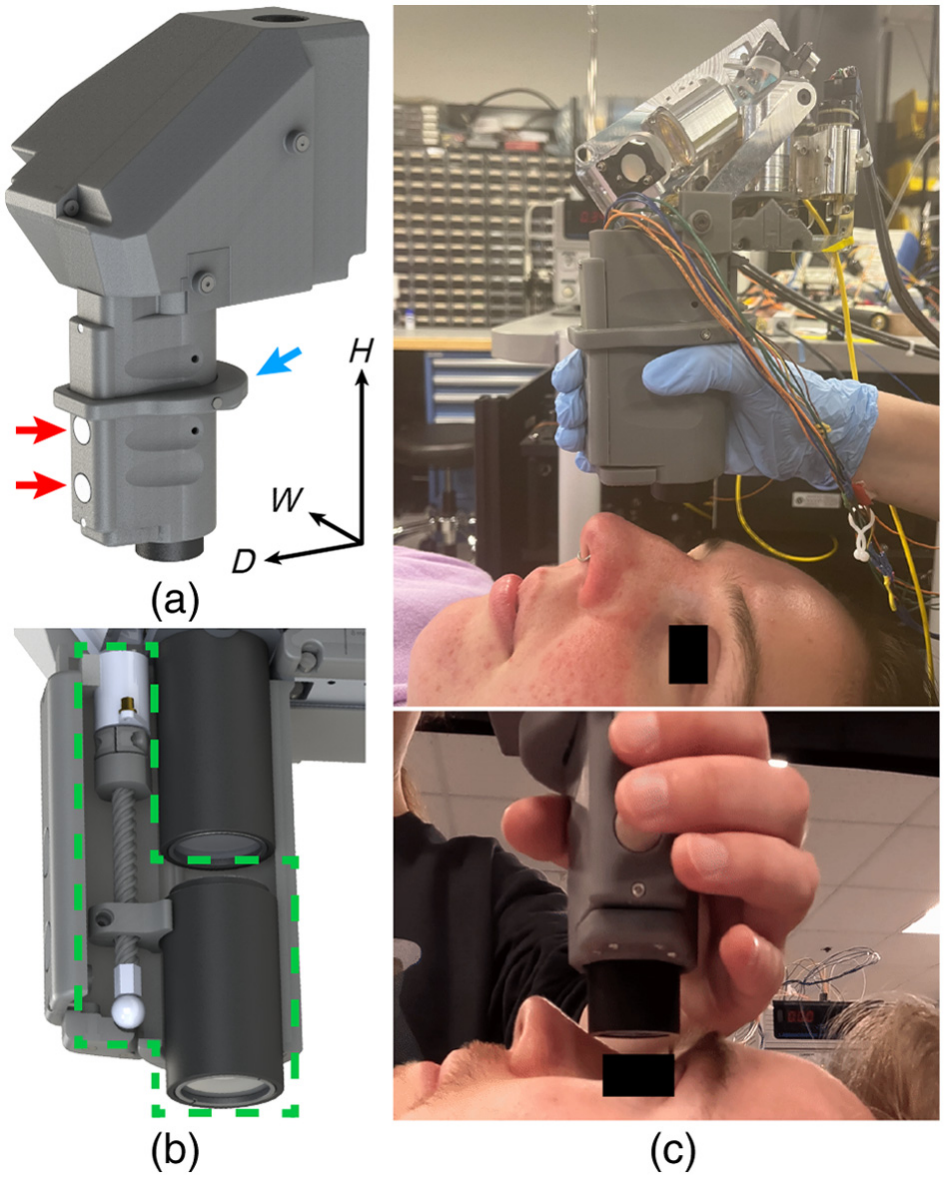
(a) Fully assembled HH-SECTR probe with ergonomic handgrip and stability brace (blue arrow) and force sensors (red arrows) for ophthalmic lens translation. (b) Motorized focusing system (dashed green box) within handgrip. (c) HH-SECTR imaging of healthy volunteers.

Multimode collection and direct detection with an avalanche photodiode leave SER signals susceptible to saturation from specular surface reflections, such as the apex of a lens surface, which precludes SER focus adjustment with tunable lenses with flat optical windows. Additionally, the use of two tunable lenses for separate OCT and SER focus adjustment would add to HH-SECTR probe weight and hardware complexity. A rapid-prototyped handgrip with a custom lightweight motorized focusing subsystem was integrated into the HH-SECTR scan head to enable parfocal adjustment of SER and OCT without tunable lenses and with minimal weight contributions to the probe. OCT and SER share the fast-axis scanner, therefore making a downstream lens group ideal for parfocal adjustment. An IC-driven bipolar stepper motor (TB6612FNG, Toshiba, Minato City, Japan; PG15S-D20-HHB9, NMB Tech. Corp., Novi, Michigan, United States) and a custom lightweight linear actuation system (18.8 g) translate the ophthalmic lens within the handgrip [Fig. 5(b)] with minimal added bulk compared to off-the-shelf translation stages. The focusing range was limited to ±10 diopters (D) (±5.5  mm of lens translation) to ensure no beam clipping at the edge of the ophthalmic relay lens aperture, which accommodates most of the ametropic population, including severe myopes and premature infants.^25^

The handgrip includes an adjustable stability brace for resting on the photographer’s thumb, purlicue, and index finger to reduce hand and arm exertion during imaging.^6^ A rapid-prototyped handgrip was chosen for (1) easy customizability to conform to a specific photographer’s hand anatomy for optimal comfort during imaging, (2) easy adjustment of the stability brace, (3) quick production and replacement if necessary to maintain clinical sterility, and (4) to minimize additional probe weight despite increased mechanical stability from metal. The handgrip was designed to mount directly to the strut, scanning lens tube, and lens tube mount on the scan head to minimize any contributions to optical beam displacement during imaging. Calibrated force sensors (FSAGPNXX1.5LCAC5, Honeywell International Inc., Charlotte, North Carolina, United States) in the handgrip placed under the photographer’s fingers allow ergonomic focus adjustment with minimal finger exertion [Fig. 5(a)] and eliminate the transfer of kinetic energy from latching mechanisms into the photographer’s hand to minimize hand motion artifacts [Fig. 5(c)].^35^ The motorized focusing system and force sensors were integrated with a microcontroller for an overall system response time of 100  μs. The motorized focusing actuator (18.8 g), force sensors (12.0 g), wiring (23.8 g), handgrip (71.5 g), stability brace (24.1 g), ophthalmic lenses (37.5 g), and lens tube (21.3 g) added a total weight of 209.0 g. When combined with the aluminum scan head, the HH-SECTR probe measured a total of 10.2  cm×14.7  cm×22.8  cm [W×D×H, Fig. 4(a)] and weighed 957 g, which is a 27% weight increase compared to the previous resin-based probe (752 g).^5^

## 3 Results

### 3.1 Optical Performance

**Fig. 6:**
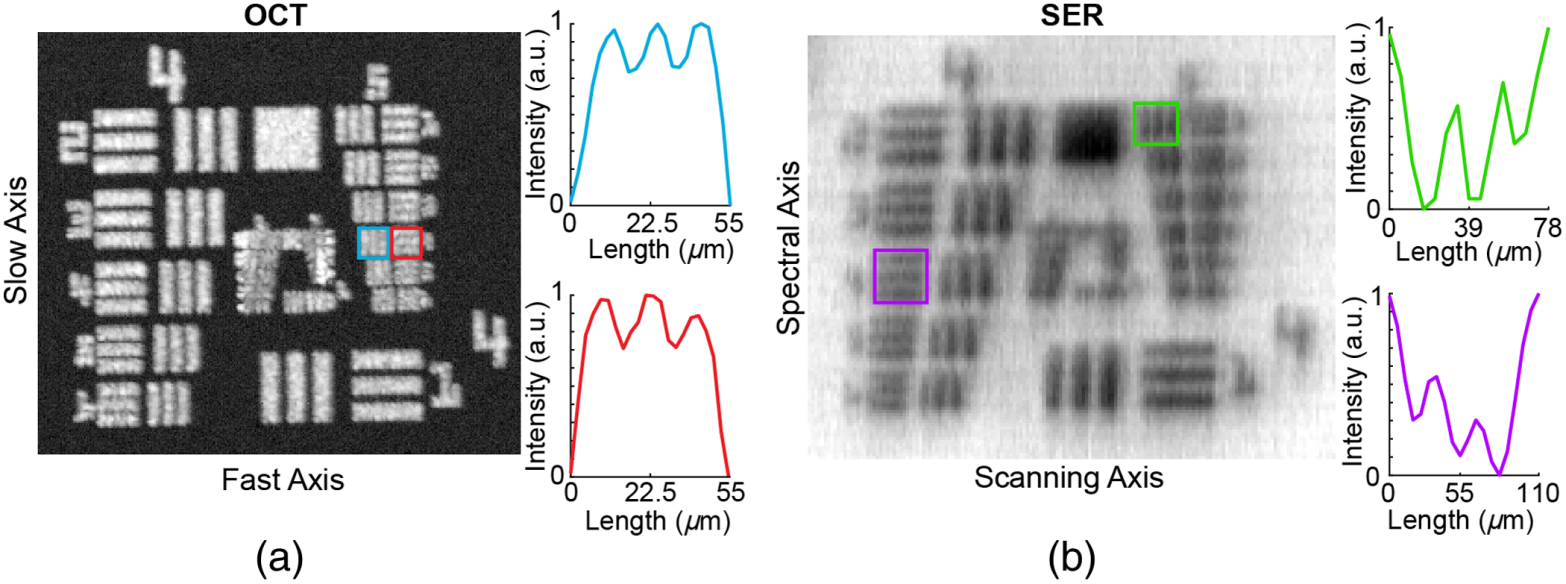
HH-SECTR resolution characterization. (a) *En face* OCT volume projection, with contrast cross-sections of group 5, element 4 for the fast (blue) and slow (red) axes. (b) SER image, with contrast cross-sections of group 5, element 1 (green), and group 4, element 4 (purple) for the scanning and spectral axes, respectively. OCT and SER were sampled at 1792×1250×1250  pixels (spectral samples per line × lines per frame × frames per volume) over a 3.9  mm×3.9  mm FOV.

HH-SECTR imaging performance was evaluated by oversampling a 1951 United States Air Force resolution chart in the IIP after the scan lens (Fig. 1, $f_{\text{scan}}$) because image quality and resolution at the retina will ultimately be limited by individual ocular aberrations. The Airy radii of the optical design for OCT and SER were 9.8  μm and 9.6  μm, respectively.^5^ Unattenuated pupil plane powers were 8.5 mW for SER and 2.5 mW for OCT. SER power was reduced to 0.01 mW to minimize reflection artifacts for resolution quantification. Figures 6(a) and 6(b) show OCT and SER, respectively, sampled at 1792×1250×1250  pixels (spectral samples × lines per frame × frames per volume) over a 3.9  mm×3.9  mm FOV. The slow and fast axes of OCT both resolved group 5 element 4 (11.05  μm). The spectrally encoded and scanned axes of SER resolved group 4 element 4 (22.10  μm) and group 5 element 1 (15.63  μm), respectively. Only the SER spectrally encoded-axis resolution performed worse than design specifications (9.47  μm).

### 3.2 HH-SECTR Optomechanical Stability

**Fig. 7:**
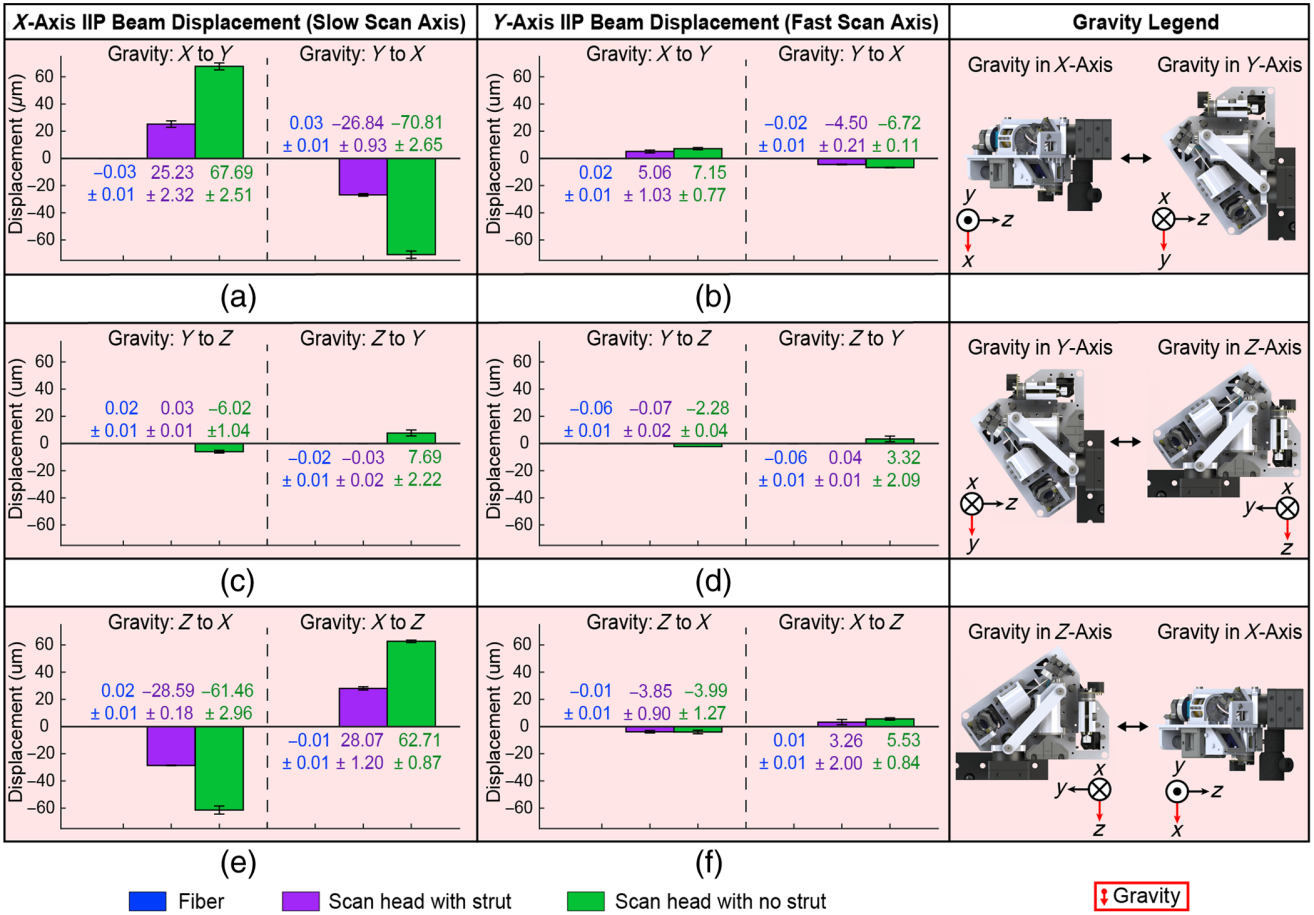
Experimental results to validate HH-SECTR scan head stability with quantified optical beam displacement. Change in lateral beam displacement is shown as the relay setup was rotated to reorient gravity (red arrow, gravity legend) between (a), (b) X- and Y-axes, (c), (d) Y- and Z-axes, and (e), (f) Z- and X-axes. The first (a), (c), (e) and second (b), (d), (f) columns display slow- and fast-axis beam displacement, respectively. Purple and green indicate displacement with and without the strut on the scan head, respectively. Blue shows the quantification of experimental noise from imaging of fiber in the relay setup.

Figure 7 shows relay stability experiment results, where beam displacement is scaled from the image (detection) plane to the object plane. Fiber face displacements indicate sub-micrometer relay stability and therefore minimal noise contributions from the experimental setup. Scan head stability results from the relay experiment indicate fast-axis single micrometer-scale optical beam stability, with a ∼2.5-fold reduction in slow-axis beam displacement from the addition of the strut. The X to Y/Y to X [Figs. 6(a) and 6(b)] and Z to X/X to Z [Figs. 6(e) and 6(f)] setups show up to 71  μm of slow-axis beam displacement without the strut, which is reduced to less than 29  μm with the strut. This was expected for these orientations since gravity is oriented orthogonally to the baseplate (X-axis), which revealed the most mechanical displacement in the FEAs. For all three setups, beam displacement was approximately equal and opposite when pivoting the relay among orientations of gravity (e.g., X to Y and Y to X), which indicates overall sustained optical and mechanical scan head stability.

The freehand stability experiment results are similar to the relay stability experiment results, with maximum beam displacements of ±23  μm and ±0.5  μm in the slow and fast axes, respectively, with the strut on the scan head, and ±35  μm and ±2  μm without the strut in the slow and fast axes, respectively. The reduced slow-axis displacement in the freehand data is attributed to the baseplate not being oriented orthogonally to the force of gravity as in the relay experiment. The residual 23  μm of slow-axis optical beam displacement with the strut had no impact on OCT or SER imaging when performing *in vivo* handheld retinal imaging.

### 3.3 Motorized Focusing in Ophthalmic Phantom

**Fig. 8:**
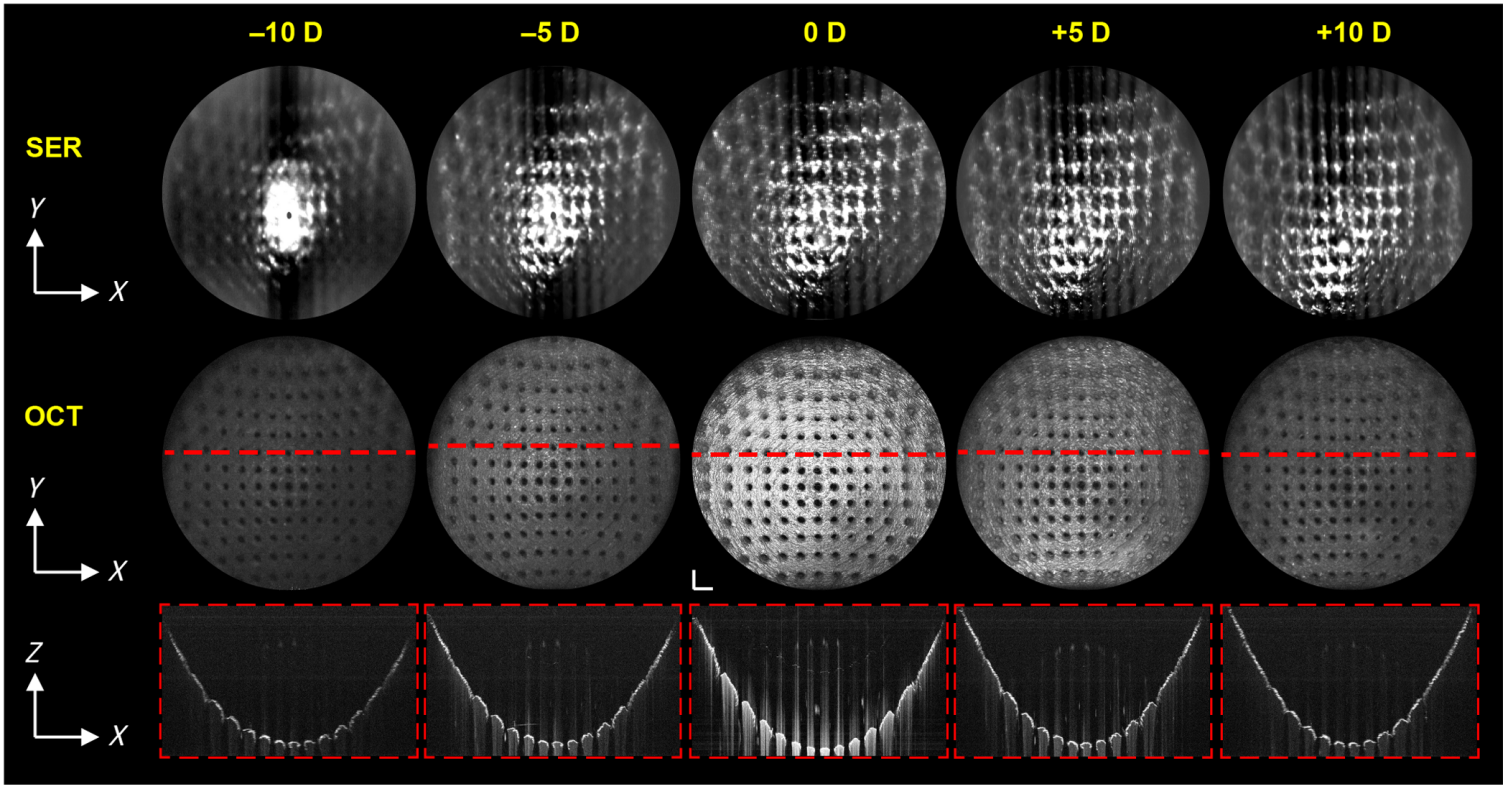
Motorized focusing on a phantom across ±10 diopters (D) with HH-SECTR. First row: *en face* SER five-frame averages. Second row: OCT projections. Third row: five-averaged OCT B-scans. Interference artifacts are visible above the phantom surface in the B-scans. Volumes were sampled in 1792×1750×1750 (spectral samples × lines × frames). Significant decreases in contrast and resolving power can be seen as the focus is shifted from 0 D. Scale bar: 2 mm.

Motorized focusing for parfocal adjustment of OCT and SER was evaluated in a stationary 0 D adult ophthalmic phantom by stepping through focal shifts over the ±10 D correction range (Fig. 8). The rapid-prototyped phantom had posterior structural features of 1-mm diameter holes spaced 2 mm apart (center-to-center). The ophthalmic lens was translated with open-loop feedback to the appropriate axial position for each focal position, which was determined by adjusting the paraxial focus of the ophthalmic eye model utilized in the optical design. SER and OCT were simultaneously sampled at 1792×1750×1750  pixels (spectral samples × lines × frames) for a frame rate of 228.57 Hz and a volume acquisition time of 7.66 s. Significant decreases in contrast and resolving power can be seen for SER and OCT as the focus is shifted from 0 D. Saturation artifacts from strong backscattering and specular reflections at the air-to-plastic interface in the phantom are visible on *en face* SER and cross-sectional OCT (Fig. 8). These artifacts were not observed *in vivo* retinal images.

### 3.4 *In Vivo* Retinal Imaging and Motorized Focusing

**Fig. 9:**
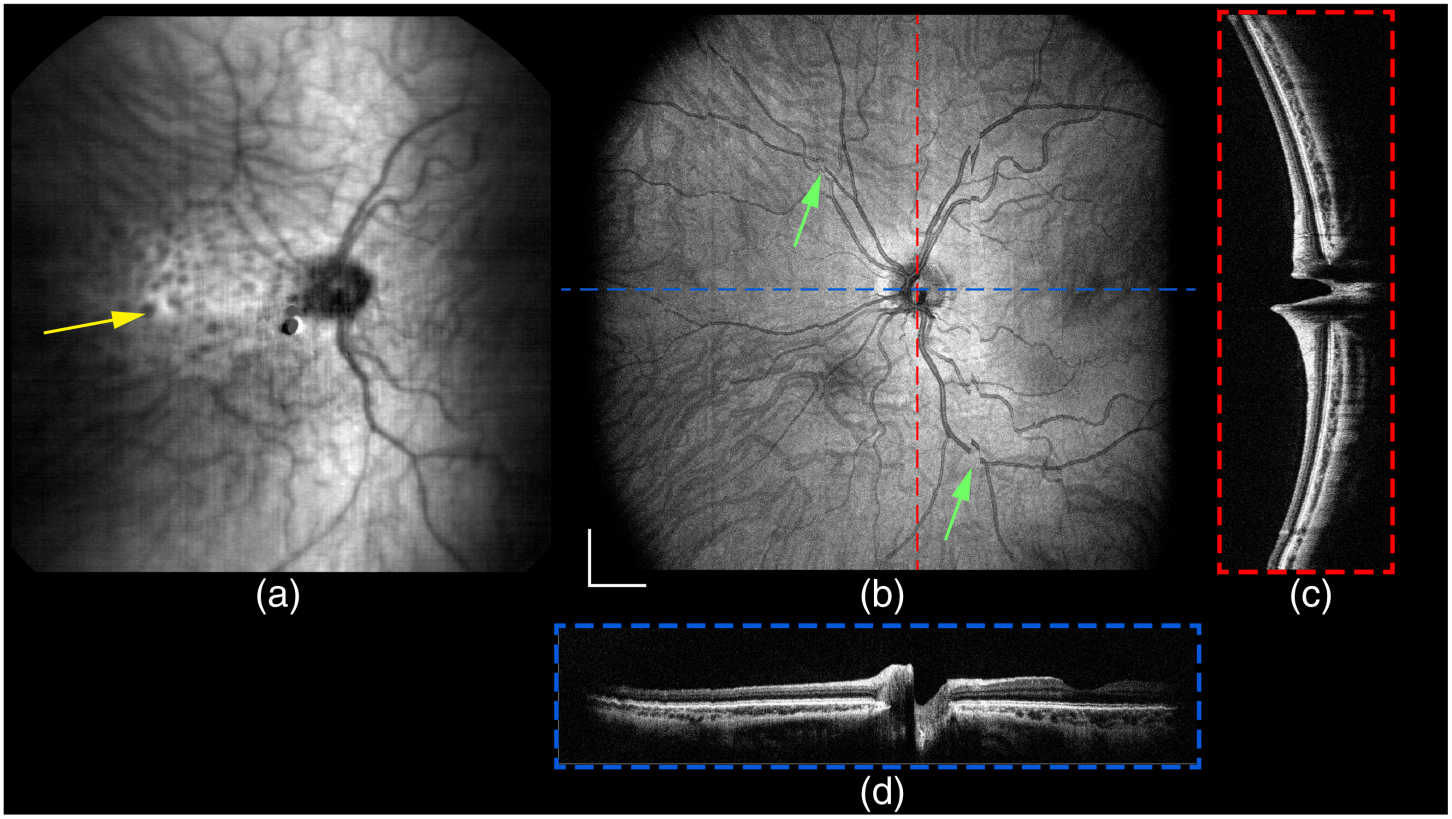
Widefield *in vivo* handheld retinal imaging with HH-SECTR (Video 2). (a) Five-averaged SER frame, (b) OCT projection, and (c) fast and (d) slow axis five-averaged OCT B-scans. The volume was sampled with 1792×1750×1750  pixels in 7.66 s with a frame rate of 229 Hz. SER data shows back-coupled reflections from the volunteer’s cornea and prescription contact lens (yellow arrow). Uncorrected ocular motion artifacts are visible in the OCT *en face* projection (green arrows). Scale bar: 1.5 mm. (Video 1, MP4, 8.25 MB [URL: https://doi.org/10.1117/1.JBO.29.7.076006.s1]).

**Fig. 10:**
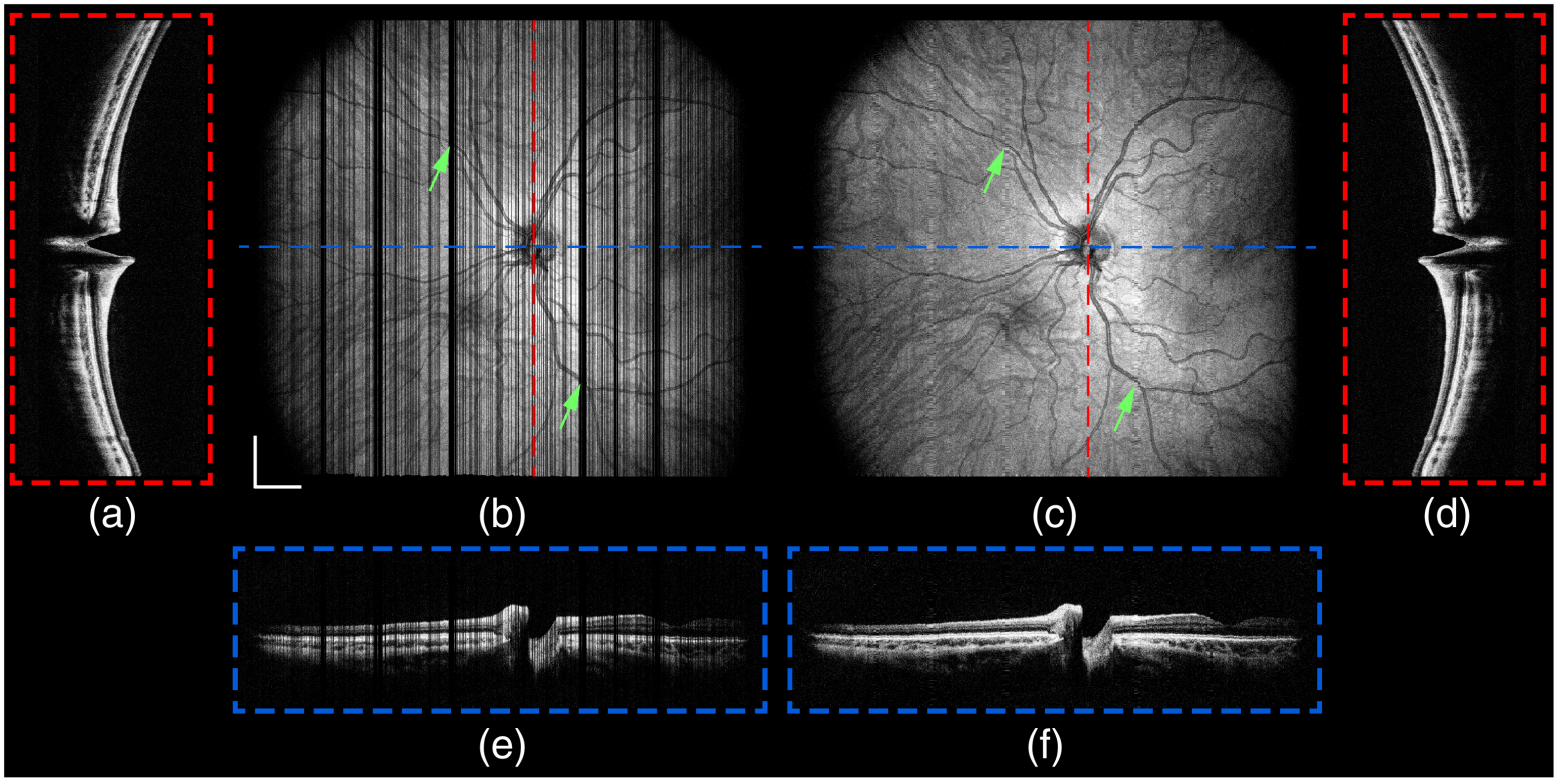
Motion correction with SER retinal tracking and subsequent interpolation of the widefield *in vivo* retinal OCT volume from Fig. 9. (a) Fast-axis five-averaged B-scan, (b) projection, and (e) slow-axis five-averaged B-scan from the motion-corrected volume. (c) Projection and (d) fast- and (f) slow-axis five-averaged B-scans after interpolation of the motion-corrected volume. Green arrows correspond to exemplary motion artifacts from Fig. 9 that were (b) corrected with SER retinal tracking and (c) interpolated to fill unsampled areas. Scale bar: 1.5 mm.

**Fig. 11:**
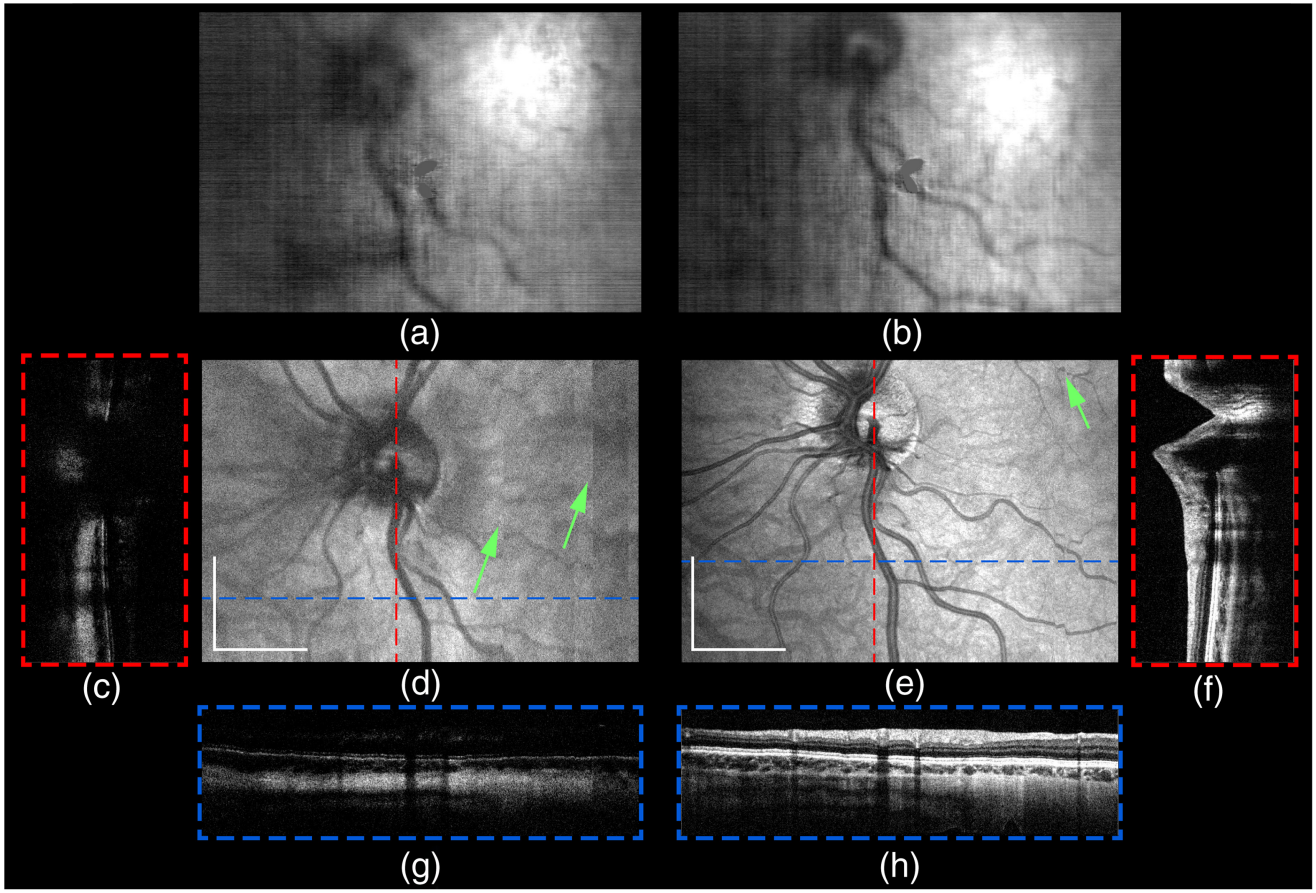
*In vivo* handheld retinal imaging of a myopic volunteer before and after focus adjustment with HH-SECTR motorized focusing. The cropped volumes were each sampled at 1792×1000×1000 for a frame rate of 400 Hz and acquisition time of 2.5 s. (a), (b) Five-averaged SER frames; (d), (e) OCT projections; and (c), (f), (g), (h) five-averaged B-scans. (b), (e), (f), (h) Focus-adjusted images show higher contrast and distinction of retinal features compared to (a), (c), (d), (g) before focus adjustment. Uncorrected ocular motion artifacts are visible in projections (green arrows). Scale bar: 1.5 mm.

Widefield *in vivo* human retinal HH-SECTR imaging and motorized focusing were performed with informed consent on a healthy adult volunteer under a Vanderbilt University Medical Center IRB-approved protocol. The volunteer was ametropic (−3 D) and wearing prescription corrective contact lenses. Powers incident at the pupil plane were 1.9 mW for OCT and 3.1 mW for SER (40 deg extended line illumination), which are below the ANSI maximum permissible exposure limit for *in vivo* human retinal imaging (ANSI Z80-36). SER and OCT were simultaneously sampled at 1792×1750×1750  pixels (spectral samples × lines × frames) for a frame rate of 228.57 Hz and a volume acquisition time of 7.66 s (Fig. 9). Lateral retinal tracking shifts from serial SER frames and axial shifts from OCT B-scans were computed using discrete Fourier transform registration and used to perform volumetric widefield data registration [Figs. 10(a), 10(b), and 10(e)].^5^^,^^34^ The registered OCT data set was interpolated to fill unsampled portions of the retina [Figs. 10(c), 10(d), and 10(f)]. HH-SECTR imaging was also performed without the patient’s corrective contacts over a 6.7×6.7  mm FOV before and after motorized focusing (Fig. 11). OCT and SER without focus adjustment (0 D position) showed low contrast and poor resolution of retinal features. OCT and SER after focus adjustment showed higher contrast and better visualized finer retinal layers and microvasculature.

## 4 Discussion

### 4.1 Summary of HH-SECTR Enhancements

This work presents an HH-SECTR system with several critical enhancements that address the limited clinical translatability and functionality of our previous design. A new engine with higher optical power was used to improve *en face* retinal SER image quality. SER and OCT imaging optics were modified to maximize optical power throughput and improve image quality. Custom SER alignment hardware was developed to improve custom double-clad fiber alignment reproducibility. A modular aluminum scan head was designed to replace the previous rapid-prototyped housing for sustained alignment during use. The stability performance of the new aluminum scan head was analyzed with FEA and subsequently optimized to minimize baseplate flexing by adding a strut. Scan head stability was experimentally quantified and demonstrated adequate stability for sustained alignment for point-of-care ophthalmic imaging. Finally, the manual zoom lens housing of the previous probe was replaced with a motorized system to improve clinical ergonomics and utility by enabling one-handed imaging and focus adjustment.

### 4.2 Spectrally Encoded Reflectometry and Retinal Tracking

The custom SER collimation fiber-bench module provided sufficient DOFs for alignment before scan head installation. Small adjustments were made after installation to ensure optimal lateral imaging resolution. SER illumination power was increased by an additional 136% at the pupil plane from our previous HH-SECTR probe by use of the higher-power source and optimization of the alignment and optical power throughput of SER relay optics.^5^^,^^28^ Additionally, the reduction of specular reflections using polarization optics, despite reducing SER power throughput, will improve *en face* aiming and registration capabilities for motion correction.^5^

Anisotropic lateral resolution is inherent to the SER optical design. The DPSL was optimized for OCT resolution with some aberrations canceling between the forward and reflected paths. Single-pass aberrations reduce resolution performance across the spectrally encoded axis in SER.^5^ Anisotropic lateral resolution does not significantly impact the real-time aiming or registration utilities of SER. However, spectrally encoded-axis aberrations can be corrected with a custom SER objective to achieve improved imaging resolution. Only the SER spectrally encoded-axis resolution performed worse than design specifications (9.47  μm). This may be attributed to mechanical tolerancing of the DPSL spacers or slight misalignment between the SER beam and the DPSL. The spectrally encoded dimension of the SER FOV is set by the illumination bandwidth, grating pitch, and system magnification. In HH-SECTR, the spectrally encoded FOV was optimized to span the retinal vascular arcades when centered on the optic nerve to ensure sufficient vascular features are consistently visible for aiming and registration.^5^

SECTR registration applies lateral shifts measured from serial SER frames to motion-correct each corresponding OCT B-scan within a volumetric dataset. Slow-axis motion necessarily results in regions of oversampled or missing data, which can be resampled or interpolated, respectively. Overlapping regions across multiple volumetric datasets can be mutually co-registered to fill in regions of missing data, perform averaging to improve the signal-to-noise ratio, or extend the imaging FOV by mosaicking.^5^^,^^28^

### 4.3 Robust Hardware

Despite the presence of rapid-prototyped HH-OCT probes in literature, little quantitative data is demonstrated for the efficacy of implementing rapid-prototyped design materials. Simpler resin-based probe designs that incorporate only OCT imaging optics may have less noticeable mechanical stability issues, unlike our more complex HH-SECTR probe, which accommodates both OCT and SER imaging optics. However, with more rigorous design and manufacturing, the use of aluminum and a modular design approach allowed us to prototype an HH-SECTR probe with a 40-fold improvement in mechanical stability compared to our previous resin-based probe, which is competitive with other HH-OCT probes in form factor.^6^^–^^8^ The hardware design and prototyping methods utilized for our HH-SECTR probe can be applied beyond point-of-care ophthalmic OCT to any application where compact and robust form factors are needed for free-space optical hardware. Quantitative design methods, such as topology optimization, may be implemented in future designs to reduce the weight of machined HH-SECTR components while preserving mechanical stability.^36^ However, optical elements account for a significant portion of total device weight (335.5 g, 35.1%). Aside from the DPSL, our current design is comprised entirely of stock optics to reduce cost. An optical redesign exclusively using optimized custom lenses may further reduce overall weight.

The use of rapid-prototyped resin for the handgrip, as opposed to the use of metal and its stability benefits, was deemed necessary to (1) minimize probe weight, (2) provide a customizable form factor for a photographer for optimal comfort during imaging, and (3) facilitate easy replacement for clinical sterility. The rapid-prototyped handgrip was not included in the stability experiments to minimize experimental complexity and to isolate stability measurements to the aluminum scan head. No stability issues were observed from the use of the rapid-prototyped handgrip during HH-SECTR imaging, but custom metal supports could be designed and integrated with the rapid-prototyped handgrip in the future at the expense of added weight.

### 4.4 HH-SECTR Stability Validation

Correlating the impact of mechanical displacement on optical performance proves complex due to the intricacy of analysis methods. The FEA methods utilized in this work exclusively analyzed material deformation under the force of gravity, did not simulate nuanced mechanical factors such as kinematic spring tension, and did not include optical performance assessments. While more complex methods with integrated optical-optomechanical analyses may prove more robust in future research, the methods utilized in this research allowed us to achieve an optimal HH-SECTR scan head design with theoretical single-micrometer mechanical stability by adding a simple strut. Methods to directly measure mechanical displacement prove difficult to implement, so OCT optical beam displacement was chosen to experimentally assess stability. The OCT optical path spans the majority of the scan head, and subsequent beam displacement proves representative of the compounding effects of any mechanical displacement across the scan head. As demonstrated by the stability experiment results, 1  μm of theoretical mechanical displacement in the FEA results of the optimized scan head with the strut resulted in 23  μm of measured optical beam displacement, which corroborates the need for high mechanical stability to maintain sustained alignment of free-space optical components hardware.

SER and OCT are co-aligned with a 50-μm beam separation and demagnified by two to the pupil for a subsequent 25  μm separation.^5^ In consideration of the beam separations and the amount of OCT beam displacement measured in the stability experiments, we estimate a maximum theoretical displacement of <100  μm between SER and OCT fields at the retinal plane, which does not impact real-time *en face* aiming with SER. Additionally, since OCT motion correction with SER is based on relative motion between sequential SER frames, and the relay experiment stability data indicates sustained beam position when the aluminum HH-SECTR scan head is held at a stationary position, slight FOV offsets will not impact registration of corresponding OCT B-scans. For these reasons, SER beam displacement was not quantified and compared to OCT beam displacement.

Both stability experiment results demonstrate fast-axis single-micrometer optical beam stability in the scan head, while the tens of micrometers of slow-axis beam wander is likely a compounding effect from baseplate flexing across the scan head. In application to clinical handheld imaging, this amount of lateral beam displacement is insignificant compared to millimeter-scale motion that occurs during imaging from photographer hand tremor (8 to 12 Hz) and patient eye drift and saccades (≤150  deg/s).^2^^–^^4^ Additionally, the relay experiment stability data indicates sustained beam position and, therefore, sustained optical alignment when the probe is held at any stationary position, which is critical for longitudinal clinical use without constant probe realignment. Conclusively, the experimental stability demonstrated by the aluminum HH-SECTR scan head is more than adequate for robust point-of-care OCT imaging.

### 4.5 Motorized Focusing

Focus adjustment is essential for diagnostic accuracy with OCT imaging in ametropic patients. As discussed in Sec. 2.7, SER signals are easily susceptible to saturation from specular surface reflections, which precludes SER focus adjustment using tunable lenses. If a tunable lens was placed after the parabolic mirror collimator, specular surface reflections from the flat optical windows of the lens would saturate any backscattered retinal signal collected through the multimode inner cladding of the double-clad fiber [Fig. 1(a)]. Motorized translation of the ophthalmic lens enables simultaneous focal adjustment of SER and OCT with one focusing element, which reduces probe weight and hardware complexity. Small optical axis misalignments can result in slight FOV shifts and distortion during focus adjustments, but these were not observed in this study. The motorized focus adjustment and handgrip-integrated focus control, while inherently photographer-driven with image quality judged by the photographer, alleviates manual lens translation by the photographer or a dedicated operator. We predict this will reduce overall imaging session time and improve clinical ergonomics. Maintaining working distance during *in vivo* imaging posed minimal challenges to the photographer with motorized focus adjustment. Closed-loop feedback for real-time image quality assessment may enable automated focus detection and tracking to facilitate even more ergonomic clinical workflow in point-of-care OCT imaging.^37^^,^^38^

## 5 Conclusions

This research presents HH-SECTR with improved *en face* SER imaging, a robust aluminum form factor for clinical translation, and one-handed motorized focus adjustment capabilities. Our design methods resulted in micron-scale optical-mechanical stability in our HH-SECTR probe, and we used it to perform point-of-care *in vivo* OCT imaging and volumetric motion correction. We predict our HH-SECTR system and synergistic technologies will benefit point-of-care OCT ophthalmic diagnostics.

## Supplementary Material





## Data Availability

Data from this work is available upon request.
